# Medicinal plants used in the traditional treatment of diabetes in Ksar Elkebir Region (North-Western Morocco)

**DOI:** 10.11604/pamj.2022.42.319.32572

**Published:** 2022-08-29

**Authors:** Ibrahim Hinad, Youssef S’hih, Aboubaker Elhessni, Abdelhalim Mesfioui, Moulay laarbi Ouahidi

**Affiliations:** 1 Laboratory of Biology and Health, Faculty of Sciences, Ibn Tofail University in Kenitra, Kenitra, Morocco

**Keywords:** Traditional medicine, medicinal plants, diabetes, Morocco

## Abstract

**Introduction:**

the number of people with diabetes continues to increase worldwide. In Morocco, two million adults are estimated to be diabetic in 2018. The Moroccan population is known for the use of medicinal plants and natural recipes for the treatment of chronic diseases including diabetes. The present study aimed to make an inventory of plant species used in folk medicine for the treatment of diabetes mellitus by diabetic patients in Ksar Elkebir City (North-west, Morocco).

**Methods:**

an ethnobotanical study was carried out among 250 diabetic patients by means of a semi-structured questionnaire by direct interviews.

**Results:**

a total of 29 species of plants belonging to 23 families were identified. The most represented families were Lamiaceae, Apiaceae, Asteraceae, fabaceae, and Lauraceae. While the most frequently cited plant species were Olea europaea l, Trigonella foenum graecum l, Origanum compactum benth l and salvia officinalis l. Leaves were the most used part of plants and the decoction was the most cited mode of preparation used by the population included in the study.

**Conclusion:**

people with diabetes in Ksar Elkebir Region use a variety of herbal remedies in several ways to treat diabetes. This result can be an important database for the following studies to confirm the efficiency of these plants in vitro and in vivo.

## Introduction

Diabetes mellitus is a metabolic disorder characterized by chronic hyperglycemia resulting from defects in insulin secretion, insulin action, or both. Diabetes mellitus may give trademark side effects, for example, thirst, polyuria, obscuring vision, and weight reduction. In it's most extreme structures, ketoacidosis or non-ketotic hyperosmolar may create and prompt daze, unconsciousness, and without effective treatment, death [1]. An estimated 463 million adults aged 20-79 years are currently living with diabetes and the total of number is anticipated to ascend to 700 million by 2045 [2]. The number of deaths resulting from diabetes and its complication in 2019 is assessed to be 4.2 million [3]. In Morocco, diabetes mellitus is one of the most widely recognized metabolic illnesses, there were over 1.6 million cases of diabetes in 2017, and it will rise to around 2.7 million in 2045 [2], and two million adult Moroccans are estimated to be diabetic, 50% of whom are unaware of their disease and each year 35000 to 40,000 new cases are notified. Thus, the number of diabetic children is estimated at more than 15000 [4]. The use of medicinal plants as a form of treatment has a long history in human history [5]. Nowadays, there is a growing interest among the public in the use of therapeutic plants, owing to patients' preference for natural products and the fact that they are inexpensive and widely available. This is especially important because diabetes places a significant financial burden on medical services and national economies [6]. According to the World Health Organization (WHO), more than 80% of the world's population, particularly in developing countries, gets their essential medical needs from medicinal herbs [7]. Indeed, traditional medicine in North Africa is a unique blend of indigenous local traditions, Christian, Islamic, Arabic, and other African customs; additionally, North Africa has a diverse climate and terrain, ranging from the Mediterranean in the north to the desert or semi-desert in the south. As a result, traditional medicine has a distinct practice throughout the region [8]. Morocco is known for it is plant diversity; in fact, it is home to 4200 of the 5000 species and subspecies recorded in North Africa [9]. The Moroccan population has been using plants for the treatment of various diseases since prehistoric times, and patients continue to rely on traditional medicine despite the development of pharmaceutical drugs [10-13]. Local folk medicine keeps being a significant source of remedies for primary medical care [14,15]. The analysis of Moroccan medicinal literature shows that documentation of local medicinal plants is divided and dissipated, so it is important to study it thoroughly [11,13,16]. In order to identify the medicinal plants used in the traditional treatment of diabetes, we conducted an ethnobotanical survey in the city of Ksar Elkebir in Northern Morocco. This survey also allowed us to study the profile of diabetics and their attitudes towards herbal medicine.

## Methods

**Subject area:** the city of Ksar Elkebir is situated in North-Western Morocco. It´s proximity of the Atlantic and the Mediterranean, 30 and 90 km respectively, gives rise to a Mediterranean climate with Atlantic influences. The summer season is characterized by high temperatures partly offset by oceanic influence. Ksar Elkebir is the most populated city of the province of Larach, at the time of the census of 2018 it counted 126,817 inhabitants (census 2018).

**Ethnobotanical survey:** the survey was carried out in Ksar Elkebir city (34°59' 56'' Nord 5°54' 10'' west, at 620 m of altitude). The survey involved 250 diabetic people, including 200 who visited the Khakhali Urban health center and 50 diabetics outside the center. The data collection was based on the interview method with translation of the questions into local language (Darija). Before starting the interview, the subjects were informed about the objective of the study, and they all cooperated voluntarily without any payment. The floristic list was established after the identification and verification of the samples; such identifications were done in collaboration with professor L Zidane (Faculty of Sciences, University Ibn Tofail in Kenitra). Scientific names were determined according to the plant list (TPL). The plants listed were collected from herbalists in the city of Ksar Elkebir and a voucher specimen of all plants identified were deposited in herbarium of the Laboratory of Biotechnology and Valorization of the Natural Resources, Faculty of Sciences, Ibn Tofail University, Morocco.

**The content of the questionnaire:** the formulary of the survey included the following parameters [12]: i) sociodemographic characteristics of diabetic patients: age, level of education, weight, type of diabetes, gender, physical activity, socio-economic level and duration of illness; ii) use of medicinal plants in the treatment of diabetes; iii) the source of provisioning their medical plants; iv) the reasons for using medicinal plants; v) name of the drug used: botanical, vernacular name, parts used and mode of preparation; vi) the results of their phototherapy and their attitudes towards therapy and medication.

**Data analysis:** the results reported on the questionnaire were entered and listed in a Microsoft Excel database and analyzed to determine the proportions of the different variables. These data were analyzed in a comparative and descriptive manner. In addition, data was analyzed using relative frequency of citation (RFC) [17]; frequency of citation= FC\N (0< RFC<1). Frequency of citation (FC) and N represent the number of participants in the survey.

**Ethical statement:** the participation in the filling of the questionnaires was voluntary, the personal data of the patients were not taken and in the respect of their privacy.

## Results

### Description of diabetic population

**Sociodemographic profiles of the diabetic patients:** the age extremes of the diabetics surveyed range from 30 to 77 years old; 4% of patients were at least young (between 30 and 40 years old), 17.6% were between 40 and 50 years old, 48% were between 50 and 60 years old, 26.4% were between 60 and 70 years old and 4% were over 70 years old. Concerning the school level, 46% of patients surveyed were illiteracy, the rest are divided between primary schooling (42%), and secondary schooling (8% with 2% college and 6% qualifying), and only 4% of diabetics had higher education levels. The minimum weight of the population concerned by this study is 60 kg while the maximum value is 140 kg but 74% of this population has a weight between 80 and 120 kg. Ten (10%) of patients have a weight between 60 and 80 kg and 16 have over 120 kg. After assembling the responses of diabetics on the type of diabetes they have, we found that 8.8 % of them have diabetes type 1, while 15.2% have diabetes type 2. However, 76% of the population surveyed ignore the type of diabetes they have. Regarding the gender of diabetics, women represented 60% compared with 40% of men 68% of the population questioned do not practice regular physical activity while only 32% follow regular physical activity. In this study, 46 percent of diabetics have low socio-economic status, 54% percent have a medium socio-economic status, and no diabetic with a high socio-economic status was found. In the population studied, the duration of the disease is very different. Indeed, there are people who have recently discovered the disease (1-4 months with 4%), while others have lived with the disease for a long time (10-20 years with 12%) and the duration of the disease most encountered in this population is that of 5-6 years (24%). Table 1 resumes the sociodemographic profiles of the patients concerned by this study.

**Table 1:** sociodemographic characteristics of diabetic patients

Variable	Subgroup	Percentage
Age	(30-40 years)	4%
	(40-50 years)	17.6%
	(50-60 years)	48%
	(60-70 years)	26.4%
	(70-77 years)	4%
Gender	Women	60%
	Men	40%
School level	Illiteracy	46%
	Primary	42%
	College	2%
	Qualifying	6%
	University	4%
Weight	(60-80kg)	10%
	(80-100kg)	44%
	(100-120kg)	30%
	(120-140kg)	16%
Type of diabetes	Type 2	15.2%
	Type 1	8.8%
	Not informed	80.76%
Physical activity	Yes	32%
	No	68%
Socio-economic level	Low socio-economic level	46%
	Medium socio-economic level	54%
	High socio-economic level	0%
Duration of illness	(1-4 months)	4%
	(1-2 years)	22%
	(3-4 years)	16%
	(5-6 years)	24%
	(7-8 years)	6%
	(9-10 years)	16%
	(10-20 years)	12%

**Use of medicinal plants in the treatment of diabetes:** of the 250 people included in the study, 150 persons (60%) have used or are still using medicinal plants to treat diabetes. However, 71.2% have never used these plants for the treatment of diabetes.

**Source of supplement:** the main source of supply of traditional medicine for our population is the experiences of others (85%), while 15% obtain traditional medicine from herbalists.

**Reasons for the use of medicinal plants:** people use traditional remedies for several reasons, 62.5% of the population studied use medicinal plants to treat diabetes because the acquisition of these remedies is easy, and the cheaper cost of the traditional remedy encourages 37.5% of our population to use them.

**Figure 1:**
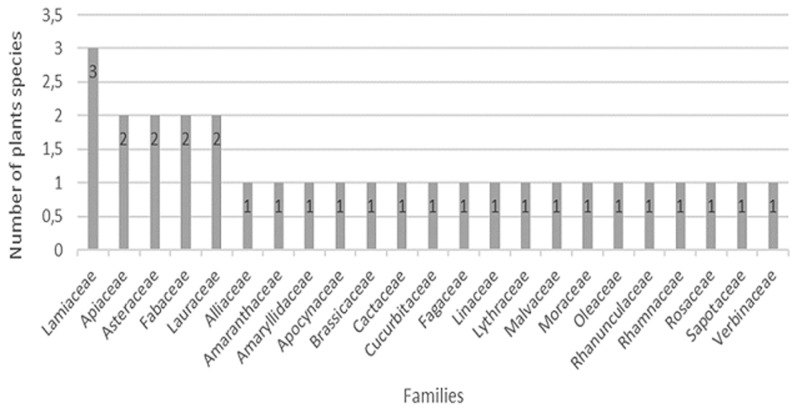
number of species in each family mentioned by the diabetic patients

**Medicinal plants used for treatment of diabetes:** the ethnopharmacological information identified confirms the diversity of medicinal plants used to treat diabetes in this region. Indeed, we arrived to identify 29 plants used in the treatment of diabetes mellitus; *Allium cepa l, Chenopodium ambrosioides l, Allium sativum l, Coriandrum sativum l, Petroselinum crispum, Caralluma europaea, Artemisia absinthium l, Inula viscosa l, Lepidium sativum l, Opuntia ficusindica l, Ucumis sativus l, Trigonella foenumgraecum l, Medicago sativa l, Quercus I, Origanum compactum benth, Salvia officinalis l, Rosmarinus officinalis l, Laurus nobilis l, Cinnamomum verum, Linum usitatissimum l, Punica granatum l, Abelmoschus esculentus l, Ficuscarica l, Olea europaea l, Nigella sativa l, Ziziphus lotus l, Prunus dulcis l, Argania spinosa, Verbena officinalis l*, Table 2 shows the distribution of plants according to their families and information on the use of these plants (scientific name, vernacular name, part used, method of preparation, and their RFC). The plants listed belong to 23 families (Figure 1). The most represented family is the Lamiaceae family with 3 species followed by the Apiaceae, the Asteraceae, the Fabaceae, and the Lauraceae family with two species. The other families are represented by a single species of plants. Figure 1 showed the number of species in each family mentioned by the diabetic patients.

**Table 2:** medicinal plants species used in the treatment of diabetes mellitus in Ksar elkebir region (Morocco)

Family name	Species	Vernacular name	Part used	Mode of preparation	RFC (%)
*Alliaceae*	*Allium cepa l*	Besla	Bulb	Raw	3,2
*Amaranthaceae*	*Chenopodium ambrosioides l*	Mkhinza	Leaves	Mac	2.4
*Amaryllidaceae*	*Allium sativum l*	Touma	Bulb	Raw	5.6
*Apiaceae*	*Coriandrum sativum l*	Lkasbour	Steam/Leave	Mac	4.8
*Apiaceae*	*Petroselinum crispum l*	Maadnous	Steam/leave	Dec	2,4
*Apocynaceae*	*Caralluma europaea l*	Deghmous	Arial part	Inf	1.6
*Asteraceae*	*Artemisia absinthium l*	Chiba	Steam/leave	Dec/Inf	3.2
*Asteraceae*	*Inula viscosa l*	Tarahla	Steam/fruit	Inf	0,4
*Brassicaceae*	*Lepidium sativum l*	Habbrchad	Seeds	Inf	0.8
*Cactaceae*	*Opuntia ficus-indica l*	Lhndiya	Steam/flower	Dec/pow	1,6
*Cucurbitaceae*	*Cucumis sativus l*	Lkhyar	Fruit	Raw	2.4
*Fabaceae*	*Trigonella foenum graecum l*	Lhalba	Seeds	Dec/pow	12
*Fabaceae*	*Medicago sativa l*	Fessa	Seeds	Pow	0.4
*Fagaceae*	*Quercus l*	Ballout	Fruit	Raw	0.4
*Lamiaceae*	*Origanum compactum benth*	Zaatar	Leaves	Inf	8,0
*Lamiaceae*	*Salvia officinalis L*.	Salmiya	Leaves	Dec/Inf	8.0
*Lamiaceae*	*Rosmarinus officinalis l*	Azir	Stem/leaves	Dec/Inf	1.2
*Lauraceae*	*Laurus nobilis l*	Orakmossa	Leaves	Inf	2.0
*Lauraceae*	*Cinnamomum verum l*	Elkerfa	Bark	Inf/dec	3.2
*Linaceae*	*Linum usitatissimum l*	Zriat elktan	Seeds	Pow	4.0
*Lythraceae*	*Punica granatum l*	Roman	Bark	Dec	2.0
*Malvaceae*	*Abelmoschus esculentus l*	Mloukhiya	Fruit	Raw/Mac	0,8
*Moraceae*	*Ficus carica l*	Lkarmous	Leaves	Mac	0,8
*Oleaceae*	*Olea europaea l*	Zitoun	Leaves	Dec	14.4
*Ranunculaceae*	*Nigella sativa l*	Haba souda	Fruit	Dec	4.0
*Rhamnaceae*	*Ziziphus lotus l*	Nbeg	Leaves	Dec/pow	0.4
*Rosaceae*	*Prunus dulcis l*	Louz	Seeds	Raw	1.6
*Sapotaceae*	*Argania spinosa l*.	Argan	Fruit	Dec	0.8
*Verbenaceae*	*Verbena officinalis l*	Louiza	Leaves	Inf	3.2

RFC: relative frequency of citation, Mac: maceration, Dec: decoction, Inf: infusion, Pow: powder

**Figure 2:**
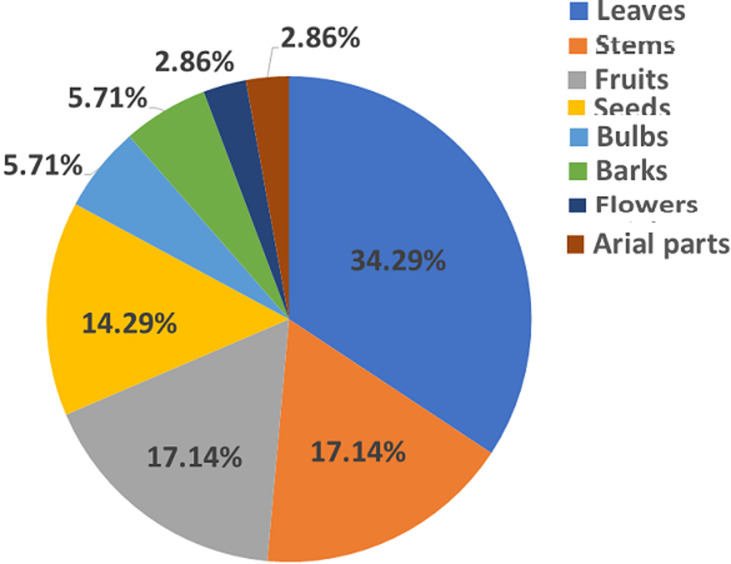
frequency of plant parts used in the treatment of diabetes mellitus

**Figure 3:**
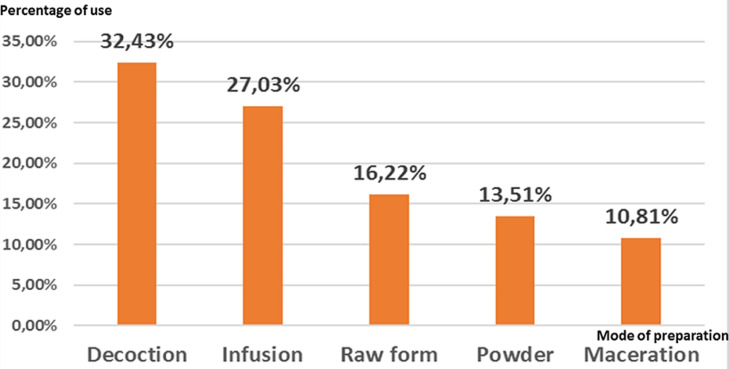
percentage of modes of preparation used by the diabetic patients

**Part used and mode of preparation of antidiabetic plants:** for the part of the antidiabetic plants, leaves were the most part used (34.29%) followed by stems and fruits (17.14%), seeds (14.29%), bulbs and barks (5.71%), flowers (2.86%), and aerial part (2.86%). Figure 2 showed the frequency of plant parts used in the treatment of diabetes mellitus. Our survey showed that decoction was the main mode of preparation of medicinal plants used in the treatment of diabetes accounting for 32.43% followed by infusion (27.03%), raw form (16.22%), powder (13.51%), and maceration (10.81%). Figure 3 showed the percentage of modes of preparation used by diabetic patients.

**Figure 4:**
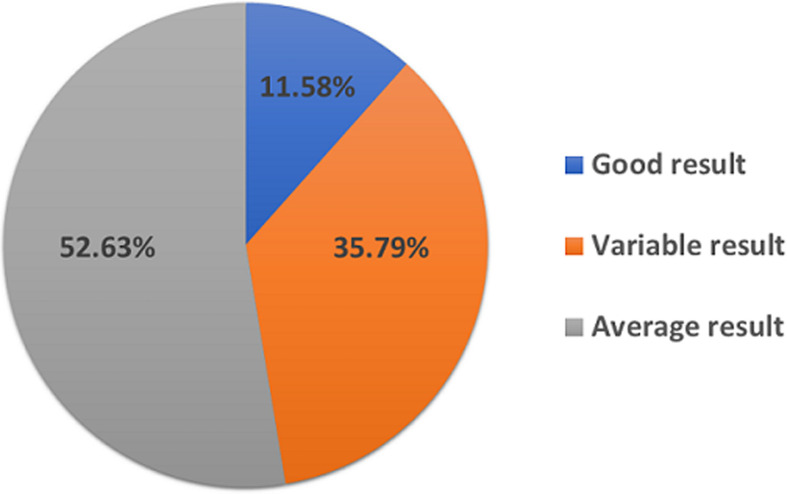
attitudes of diabetic patients towards the outcome of therapy

**Attitude towards therapy and medication:** according to the diabetic people interested in this study, the results of the therapy are not uniform, thus, 11.58% think that the therapy has a good result, 35.79% say that the result of the therapy is variable and 52.63% find that the therapy result is average (Figure 4). Regarding the attitude towards drugs, all diabetics involved in the study are satisfied with the use of drugs for the treatment of diabetes.

## Discussion

The age of the surveyed population was very different; it is between 30 years and 77 years old. Most of them (48%) belong to the age group between 50 and 60 years old. A similar study conducted by [18] showed that the age group most affected by diabetes is between 60 and 70 years old. Another survey conducted by [19] showed that the age of patients varied between 17 and 87 years, and the most represented age group was that of patients aged between 20 and 40 years old. A similar survey conducted in Fez showed that the age group most affected by diabetes included in the study was between 60 and 65 years [20]. Fouty six (46%) of patients surveyed were illiteracy. This result is in agreement with other studies in different regions of Morocco which find that illiterate diabetic patients were predominantly represented [18-21]. Due to a worldwide increase in high-calorie diets, sedentary lifestyles, and urbanization, obesity has now become an established risk factor for type 2 diabetes mellitus and or metabolic syndrome [22]. In this study, 74% of patients have a weight between 80 and 120 kg. A similar survey conducted in the town of Sidi Slimane (Northwestern Morocco) showed that the most common weight range was between 60 and 80 kg [18]. Women represented 60% of the population studied. This result agrees with other studies in other Moroccan regions [11,16,18,21]. For sports activity, 68% of the population questioned do not practice regular physical activity. This observation agrees with that found that sport is neglected by the patients and only 32.14% part take in sport [18]. Fourty six (46%) of diabetics included in the study have a low socioeconomic status while 54% have a medium socioeconomic status. A survey carried out in the town of Sidi Slimane showed that 84% of the diabetic patients belong to a medium socioeconomic level and 15.85% have a low socioeconomic and while only one person lives an easy situation [18].

This study showed that the duration of the disease is very different. Indeed, 4% have recently discovered the disease between one and four months while others lived with the disease for a long time (between 10 and 20 years). A similar study conducted in Rabat (Morocco) showed that diabetes was diagnosed in less than one year, 1-15 years, 16-30 years, and over 30 years for 14.3%, 61.2%, 22.5%, and 2% respectively [19]. This survey revealed the use of medicinal plants in the treatment of diabetes, in effect 60% of them have used or are still using herbs to treat diabetes. Many previous studies have shown that the percentage of the use of medicinal plants varies between 52% and 90% depending on the region or the area where the surveys have been undertaken [11,13,18,23]. The experiences of others are the first source of supply of traditional medicine followed by herbalists. Our study matched earlier studies that have shown that their most source was other´s experiences [18,20,24]. Patients use traditional remedies for several reasons including their easy acquisition and their price. In previous studies patients give other reasons for the use of remedies traditional; the strong use of medicinal plants is due to the strong belief of diabetic patients in their efficiency (95.86%), accessibility (2.86%) as well as their cost (1.29%) [18]. Also, patients preferred phytotherapical care justifying it by its effectiveness (62.2%) and the 30.8% left were more convinced about its availability, low cost, and almost no side effects [20].

This study allowed us to identify 29 plants used in the traditional treatment of diabetes mellitus. The most common plants used were olea europea l with the highest RFC (14.4%) followed by *Trigonnella foenum-graecum* l 12%, salvia officinalis l and origanum compactum benth l with RFC value of 8%. The most represented family is the Lamiaceae family with 3 species followed by the *Apiaceae*, the *Asteraceae*, the *Fabaceae*, and the *Lauraceae* family with two species. This result agrees with previous studies which showed that Lamiaceae is the most represented family in the plants used in the treatment of diabetes [16,18,19,21,25]. Leaves were the most plant part cited by the patients; this result agrees with previous studies that have reported leaves as the mainly used plant parts in the treatment of diabetes [16,19,26,27] in Morocco, in Algeria [25,28,29], in Turque [30,31], in South Africa [32,33], in Cameroon [34]. In India [35,36], in Bangladesh [37]. About the mode of preparation, decoction was the frequency mode of preparation used in the preparation of medicinal plants. This result is in agreement with other ethnobotanical studies where decoction was the most frequent mode of preparation [16,21,25,27].

## Conclusion

The present study reveals that traditional ethnobotany practices still play an important role in Ksar Elkebir Region. This study interested 250 people diabetics, 60% of whom have used or are still using medicinal plants to treat diabetes mellitus. The survey allowed us to identify 29 species of plants called anti-diabetic. The most cited of which are *Olea europaea l, Trigonella foenum-graecum l, Origanum* compactum benth l and *Salvia officinalis l* through this survey we also asked the patients about their attitudes towards therapy compared with drugs and we found that 11.58% of them said that the outcome of therapy was good while 35.79% found that the outcome of therapy was variable and 52.63% found it to be average. In other said all the patients were satisfied with the drugs. The current study is a very important document to preserve knowledge on the use of medicinal plants used to treat diabetes mellitus by the population of Ksar Elkebir Region. Also, it can be used as baseline data for phytochemical, toxicological, and pharmacological studies.

### What is known about this topic

- Traditional medicine is present among the Moroccan population to treat various diseases including chronic diseases;
- Several similar surveys have been carried out in different regions of Morocco.

### What this study adds

- This study was conducted in an unstudied region of Morocco (North-western);
- This study allowed us to identify 29 medicinal plants known as anti-diabetic, their used parts and their mode of preparation.
